# Improving intervention design to promote cervical cancer screening among hard-to-reach women: assessing beliefs and predicting individual attendance probabilities in Bogotá, Colombia

**DOI:** 10.1186/s12905-022-01800-3

**Published:** 2022-06-07

**Authors:** David Barrera Ferro, Steffen Bayer, Sally Brailsford, Honora Smith

**Affiliations:** 1 Southampton Business School, University of Southampton, Southampton, UK; 2 Departamento de Ingeniería Industrial, Pontificia Universidad Javeriana, Bogotá, Colombia; 3 Mathematical Sciences, University of Southampton, Southampton, UK

**Keywords:** Cervical cancer screening, Health belief model, No-show prediction, Hard-to-reach women

## Abstract

**Background:**

Despite being a preventable disease, cervical cancer continues to be a public health concern, affecting mainly lower and middle-income countries. Therefore, in Bogotá a home-visit based program was instituted to increase screening uptake. However, around 40% of the visited women fail to attend their Pap smear test appointments. Using this program as a case study, this paper presents a methodology that combines machine learning methods, using routinely collected administrative data, with Champion’s Health Belief Model to assess women’s beliefs about cervical cancer screening. The aim is to improve the cost-effectiveness of behavioural interventions aiming to increase attendance for screening. The results presented here relate specifically to the case study, but the methodology is generic and can be applied in all low-income settings.

**Methods:**

This is a cross-sectional study using two different datasets from the same population and a sequential modelling approach. To assess beliefs, we used a 37-item questionnaire to measure the constructs of the CHBM towards cervical cancer screening. Data were collected through a face-to-face survey (*N* = 1699). We examined instrument reliability using Cronbach’s coefficient and performed a principal component analysis to assess construct validity. Then, Kruskal–Wallis and Dunn tests were conducted to analyse differences on the HBM scores, among patients with different poverty levels. Next, we used data retrieved from administrative health records (*N* = 23,370) to fit a LASSO regression model to predict individual no-show probabilities. Finally, we used the results of the CHBM in the LASSO model to improve its accuracy.

**Results:**

Nine components were identified accounting for 57.7% of the variability of our data. Lower income patients were found to have a lower Health motivation score (*p*-value < 0.001), a higher Severity score (*p*-value < 0.001) and a higher Barriers score (*p*-value < 0.001). Additionally, patients between 25 and 30 years old and with higher poverty levels are less likely to attend their appointments (O.R 0.93 (CI: 0.83–0.98) and 0.74 (CI: 0.66–0.85), respectively). We also found a relationship between the CHBM scores and the patient attendance probability. Average AUROC score for our prediction model is 0.9.

**Conclusion:**

In the case of Bogotá, our results highlight the need to develop education campaigns to address misconceptions about the disease mortality and treatment (aiming at decreasing perceived severity), particularly among younger patients living in extreme poverty. Additionally, it is important to conduct an economic evaluation of screening options to strengthen the cervical cancer screening program (to reduce perceived barriers). More widely, our prediction approach has the potential to improve the cost-effectiveness of behavioural interventions to increase attendance for screening in developing countries where funding is limited.

**Supplementary Information:**

The online version contains supplementary material available at 10.1186/s12905-022-01800-3.

## Background

Cervical cancer is a preventable disease. However, in 2018, it was the fourth leading cause of cancer death among women worldwide [1]. Although the overall Age Standardized Incidence Rate (ASIR), per 100,000 women is 13.1, it ranges from 6.0 in Australia and New Zealand to 40.1 in Eastern Africa [1]. In fact, both incidence and mortality rates are associated with poverty and limited health education [2–4]. In 2018, around 84% of the cases and 88% of cervical cancer deaths occurred in poorly-resourced countries [1]. Consequently, in 2020, the World Health Organization defined a set of goals to eradicate cervical cancer as a public health problem, emphasizing the need to improve human papillomavirus (HPV) vaccination coverage and screening uptake rates [5]. Nevertheless, while in high income countries the implementation of screening and vaccination programs has been successful, for many lower and middle-income countries (LMICs) it still represents a major challenge [6–9]. In Colombia, the ASIR is 12.57 and mortality rates show geographical patterns affecting disproportionally low-income women [10].

ACS *(Acciones Colectivas en Salud)* is an outreach program designed by the Health Office in Bogotá (*Secretaría Distrital de Salud*, SDS) to increase health service utilization among hard-to-reach populations. The main idea is to improve health outcomes by engaging low-income patients with eleven preventive care strategies. In this context, some of the ACS activities are devoted to increasing early cervical cancer detection by improving Pap smear test uptake among hard-to-reach women. Every month, a group of community workers identifies women who are not complying with the screening program, visits them at home, provides basic training in cervical cancer risks, and schedules a Pap smear test for them at the nearest healthcare facility. Despite this effort, around 40% of the visited patients end up missing their appointments. Therefore, more information is required to design interventions aimed at increasing attendance levels. Indeed, behavioural interventions informed by patient beliefs about screening have been found to increase uptake rates [11]. Additionally, accurate predictions of individual no-show probabilities could improve resource allocation by identifying those patients who would benefit the most from such interventions [12].

The Health Belief Model (HBM) is a widely used conceptual framework in health behavioural research [13]. In its original version, introduced in the 1950s, the underlying theory is that the adoption of a protective health behaviour can be explained by the patient’s perceptions of their susceptibility and the severity of the “threat”, and the benefits of and barriers to the behaviour [14]. Later, the model was extended to incorporate other categories [13]. Rosenstock et al. [15], for example, proposed the inclusion of a Health Motivation category to assess the patient’s incentive to behave and maintain general good health. More recently, Champion [16] developed instruments to measure HBM constructs related to breast cancer behaviour. According to Ritchie et al. [17], Champion’s revised HBM (CHBM) has been found to explain between 25 and 89% of the variance in participation in mammography studies, in different contexts, over almost 40 years. Recent reviews on the use of the HBM to study cancer prevention behaviours can be found in [17–19].

In 2010, Guvenc et al. [20] adapted the instruments of the CHBM to assess beliefs towards cervical cancer screening. Since then, several studies have adopted the CHBM as a conceptual framework to understand cervical cancer screening behaviours. As expected, the resulting scores for each construct are highly context dependent. For example, studies using Guvenc’s scale have found susceptibility scores ranging from 2.2 in Saudi Arabia [21] to 4.8 in the USA [22]. Consequently, two recent reviews have highlighted the need to conduct local empirical research to inform public policy and design tailored interventions, particularly among marginalized communities [23, 24].

This study aims to inform the design of behavioural interventions to increase attendance levels for cervical cancer screening, among hard-to-reach low-income women in Bogotá. To achieve this, we propose a two-fold approach: cervical-cancer belief assessment and individual no-show probability prediction. A cross-sectional face-to-face survey of a random sample of ACS patients was conducted. Our analytical approach is three-fold: first, we study the reliability and construct validity of Guvenc’s scale in our study context. Next, descriptive statistics and pairwise comparison of means are used to analyse the CHBM constructs. Finally, we develop a model to predict individual no-show probabilities using the survey results, patient sociodemographic information and appointment characteristics.

## Methods

This section starts with a description of our study context. We provide basic information about the cervical screening program in Colombia and the definition of hard-to-reach women used by ACS in Bogotá. The beliefs assessment then follows, describing the survey instrument, its validation, and data collection procedure. Finally, we present the proposed modelling approach to predict individual attendance probabilities.

### Study context and sample

In Colombia the coverage of the vaccination against the Human Papilloma Virus (HPV) remains low, despite being included in the free national immunization program [25]. Therefore, cervical cancer control strategy is focused on early detection through screening. Women between 25 and 69 are eligible, following a 1–1-3 scheme. This means that screening is recommended annually and changed to a three-year interval after two consecutive annual negative results. Currently, the program is primarily based on cervical cytology and is included in the national health insurance, so no out-of-pocket payment is required when undergoing the examination [26, 27]. However, women do not receive any formal invitation to book a cytology appointment. Thus, the program relies on doctor recommendations and patient motivation. Although recent legislation recommended starting a transition to a HPV-test-based screening [27], the National Ministry of Health assessed operational barriers and decided to delay the pilot phase [28]. SDS considers a patient to be hard-to-reach if despite being eligible, she has not attended a screening appointment in the preceding year. Additionally, low-income populations are classified into four poverty levels to prioritize their participation in social programs. In this context, ACS only covers people belonging to the three most severe levels of poverty (High, Medium, and Low). Our study population are hard-to-reach women covered by ACS in Bogotá.

All items in the CHBM questionnaire used a three-point Likert scale: disagree, neutral and agree. The aim of the cross-sectional survey was to estimate the proportion of patients selecting each option. In December 2019, 43,500 hard-to-reach women were covered by ACS. In the absence of information about responses to any of the CHBM questions among this target population, the sample size was determined using an assumed proportion of 50% ‘yes’ responses to a hypothetical yes/no question, with confidence level 95% and error 2.5%. This gave a required sample of at least 1485 participants. Following a process of stratified random sampling, SDS eventually invited 1750 hard-to-reach women to take the survey. A total of 1699 women (97%) consented and SDS provided the anonymized answers. Although the women in our study population were designated hard-to-reach, they were willing to receive a home visit from the ACS team and were asked to take the survey at the end of the visit. This might offer an explanation for the high uptake, as no incentives were offered. Additionally, appointment information and socio-demographic data (i.e. age of the patient and poverty index) were retrieved from SDS information systems. Pontificia Universidad Javeriana (FID-19–107), SDS (2019EE47807) and the University of Southampton (ERGO ID 48,583.A1) granted ethical approval for this study.

### Assessing beliefs

We used the items of the CHBM questionnaire for cervical cancer screening and Pap smear test, developed by Guvenc et al. [20]. The statements were translated into Spanish and discussed with public health experts from SDS. As a result, taking into account the study context, six items were added to the list and five deleted. Hence, we used a 37-item survey (see Table 1) to assess the five constructs of the model: Susceptibility (4 items), Severity (7 items), Benefits (8 items), Health motivation (3 items) and Barriers (15 items). For data analysis, values of 1 (disagree), 3 (neutral) and 5 (agree) were assigned, following the convention in the literature [29]. Construct validity was evaluated using principal component analysis and sample adequacy was assessed with the Kaiser–Meyer–Olkin (KMO) test. Finally, reliability of the scale was examined using item-rest subscale correlation and Cronbach’s Alpha coefficients.

**Table 1:** CHBM Survey

Category	No	Statement
Susceptibility	1	It is likely that I will get cervical cancer in the future
Susceptibility	2	My chances of getting cervical cancer in the next few years are high
Susceptibility	3	I feel I will get cervical cancer sometime during my life
Susceptibility	4	I feel I will get cervical cancer sometime during my life because I have family history of cancer
Severity	5	The thought of cervical cancer scares me
Severity	6	When I think about cervical cancer, I feel worried
Severity	7	I am afraid to think about of cervical cancer
Severity	8	Problems I would experience with cervical cancer would last a long time
Severity	9	Cervical cancer would threaten a relationship with my husband, boyfriend, or partner
Severity	10	If I had cervical cancer my whole life would change
Severity	11	If I developed cervical cancer, I would not live longer than 5 years
Benefits	12	I want to discover health problems early
Benefits	13	Maintaining good health is extremely important to me
Benefits	14	I look for new information to improve my health
Benefits	15	I feel it is important to carry out activities which will improve my health
Benefits	16	Having regular Pap smear tests will help to find changes to the cervix, before they turn into cancer
Benefits	17	If cervical cancer was found at a regular Pap smear test its treatment would not be so bad
Benefits	18	I think that having a regular Pap smear test is the best way for cervical cancer to be diagnosed early
Benefits	19	Having regular Pap smear tests will decrease my chances of dying from cervical cancer
Motivation	20	I eat well-balanced meals for my health
Motivation	21	I exercise at least 3 times a week for my health
Motivation	22	I have regular health check-ups even when I am not sick
sBarriers	23	I am afraid to have a Pap smear test for fear of a bad result
Barriers	24	I am afraid to have a Pap smear test because I don’t know what will happen
Barriers	25	I don’t know where to go for a Pap smear test
Barriers	26	I would be ashamed to lie on a gynaecologic examination table
Barriers	27	Undergoing a Pap smear test takes too much time
Barriers	28	Undergoing a Pap smear test is too painful
Barriers	29	Health professionals performing Pap smear tests are rude to women
Barriers	30	I have other problems in my life which are more important than having a Pap smear test
Barriers	31	I am too old to have a Pap smear test regularly
Barriers	32	Undergoing a Pap smear test is too uncomfortable
Barriers	33	I think that having a regular Pap smear test is required only if one has an active sexual life
Barriers	34	My religion does not allow me to undergo a Pap smear test
Barriers	35	Preparing for a Pap smear test can be inconvenient for me
Barriers	36	Undergoing a Pap smear test can cause problems with my partner
Barriers	37	I am too young to have a Pap smear test regularly

Community workers collected data, at the end of home visits, between January and February 2020. Before data collection started, training took place in eight workshops with 280 community workers. During these workshops, the research project was presented and items of the instrument were analysed. As part of their enrolment process with SDS, community workers were previously trained in data collection, interaction with vulnerable communities and techniques to discuss health-related topics. Due to security concerns, it was decided that a printed version of the instrument should be used with each participant. Raw data were stored and anonymized by SDS. We used descriptive statistics to assess beliefs about cervical cancer screening. Pairwise comparison of means was performed to examine the effect of the participants poverty levels on each construct of the CHBM.

### Predicting individual no-show probabilities

We analysed two data sets. First, SDS provided anonymised data from the 1699 surveyed patients (dataset 1). Table 2 presents the list of variables collected by ACS program managers, grouped into patient and appointment characteristics. These variables have been found to have good predictive value for medical appointment attendance [30]. Five of these variables (age, lead time, month, and day) were previously used to model no-show behaviour for preventive care appointments in Bogotá [31]. We also retrieved data from historical administrative records (dataset 2) relating to appointments scheduled for 23,384 women between 2017 and 2019 as part of the ACS program. Further details of the two datasets can be found in Additional File 1: Table S1, where it can be seen that the sociodemographic profiles of the women in both datasets are similar.

**Table 2:** Variables used for the LASSO model

Category	Variable	Description	Highest no-show		Lowest no-show	
			Data set 1	Data set 2	Data set 1	Data set 2
Patient	Age	Age of the patient at the time of the appointment (years)	[30–49]	< 30	> 59	> 59
			41%	51%	24%	29%
	Poverty	Poverty level indicator defined by the national planning department	High	High	Medium	Low
			46%	40%	34%	34%
Appointment	Lead time	Elapsed time between the date of the home visit and the appointment date (days)	≥ 16	≥ 16	[8–15]	[8–15]
			39%	38%	30%	35%
	Month	Month in which the appointment was scheduled	January	February	February	January
			42%	39%	33%	36%
	Day	Day of the week in which the appointment was scheduled	Saturday	Saturday	Monday	Sunday
			38%	44%	35%	22%

The methodology is described in detail in [31] and is summarised briefly here. For age and lead time we used decision trees to build categorical variables aiming at increasing model stability [32]. Additionally, one-hot encoding was used to represent all the variables in the models. To improve interpretability, we performed variable selection using a LASSO (Least Absolute Shrinkage and Selection Operator) regression model [33]. In cases with high correlation between independent variables, this model has been found to select only the best predictors and set the coefficients of the other variables to zero, avoiding multicollinearity problems [34]. Finally, we randomly generated training (70%) and test sets (30%). Table 1 also shows the categories with the highest and lowest no-show rates, for each variable in each data set. For example, while in the data set 1 the patients between 30 and 49 years old have the highest no-show rate (41%), in the data set 2 the patients younger than 30 years old have a no-show rate of 51%. Detailed information about the samples, frequencies, and attendance levels for both data sets are provided in Additional file 1: Table S1.

To quantify the linear relationships between each variable and the no-show probability, we fitted a LASSO regression model. This model was proposed to overcome the accuracy and interpretability limitations of ordinary least-squares regression [33] and has been widely used to predict appointment attendance [30]. In future, SDS will use individual no-show probabilities to classify patients into three groups: A, B and C. While patients in groups A (at high risk of no-show) and B (at medium risk) will receive different behavioural interventions, patients in group C (low risk) will not receive any intervention as they are likely to attend anyway. Therefore, we needed to select two cut-off points. This process is called cut-off point tuning and is based on ROC performance indicators [35]. Consequently, the performance of the model was assessed using the average Area Under the Receiver Operating Characteristic (AUROC) score. This score ranges from 0 to 1, and can be interpreted as the average sensitivity of the classification considering all possible specificities [35].

We analysed average coefficients over 100 experiments. For each group of ten experiments, we randomly divided the data into ten groups, using nine for training and the other one for testing. Then, the testing group was iteratively changed. When this procedure is repeated 10 times, it is called a 10-by-10 cross validation process. Additionally, a parametric analysis was carried out to determine the penalty constant of the model. We decided to use the constant that maximizes AUROC score while maintaining the minimum possible number of variables. Scikit-Learn's logistic regression was used in our analysis, setting the alpha value to 0 [36].

To quantify the impact on accuracy, we conducted three experiments. For model 1, we used the variables presented in Table 1 for the surveyed patients (*n* = 1,699). For model 2, we used the same data set and included responses to the 37-item survey instrument. For model 3 a sequential approach was used as follows. First, we trained a model with the variables presented in Table 1, using information from Pap smear test appointments that were scheduled between 2017 and 2019 (dataset 2, *n* = 23,384). We hypothesized that by using these historical data the model would be better able to identify patterns of attendance. With this model, we predicted the no-show probability for each patient in the survey data set. Then, a second model was fitted using the first model prediction and the 37 items in the survey.

## Results

We present our results organized in three sections. Firstly, an assessment of the beliefs is presented. Then, the LASSO regression results are summarized. We use average odds ratios (OR) to quantify the impact of each variable on the attendance probability. Finally, the performance of the prediction approach is assessed. We analyse the added value, in terms of AUROC score, of using a sequential approach to predict individual attendance probabilities.

### Assessing beliefs

**Fig. 1:**
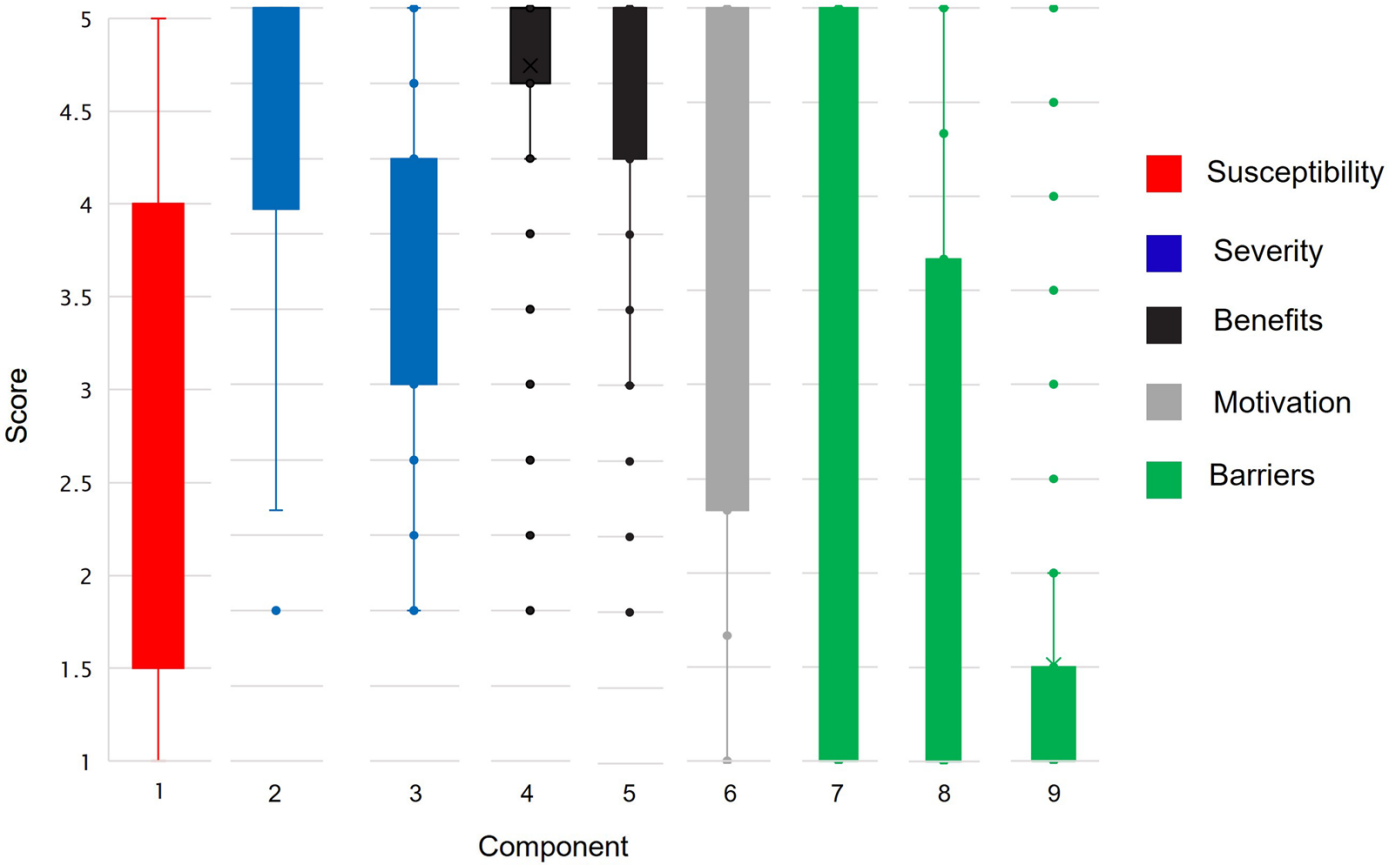
Distributions of the scores by component

Figure 1 presents the distribution of the scores for the 37 items, grouped into nine components. We provide detailed results of the item reliability analysis and construct validation for the instrument in the Additional File 2. Response frequencies by item are provided in Additional File 1: Table S2. The average susceptibility score is 2.86, with 3.83 being the highest observed value (statement 1). When presented with the statement “*It is likely that I will get cervical cancer in the future”,* 56% of the participants agreed. However, judging by the other three items in the category, most of the participants showed a low perceived susceptibility. More than 40% of the participants disagreed with statements 2, 3 and 4. Similarly, only one component is identified for the heath motivation category. The average score for health motivation is 3.52, with items ranging from 3.05 to 4.03 on average. While 71% of the participants agreed with statement 20 *“I eat well-balanced meals for my health”*, only 44% reported that they exercise at least 3 times a week for their health (statement 21).

Figure 1 also shows that severity items were grouped into two components (2 and 3). The average score for component 2 is 4.27 and it includes items 5, 6 and 7. These items are all related to feeling anxiety about the idea of cervical cancer. On average, 78% of the participants agreed with these three statements. However, severity score decreases when participants are asked about possible consequences of the disease. The average score for component 3 is 3.37, with values ranging from 2.82 to 4.14. Finally, statements 9 and 10 score bellow 3.0. While 42% of the participants disagreed with the statement *“Cervical cancer would threaten a relationship with my husband, boyfriend or partner”*, 43% provide the same answer for the statement *“If I developed cervical cancer, I would not live longer than 5 years”.*

Barriers statements are grouped into components 7, 8 and 9 with average scores of 2.63, 2.34 and 1.51, respectively. Component 7 includes statements 23 and 24 both related to being afraid to have a Pap smear test, either because of a possible bad result or because they do not know what might happen. Both statements have similar distribution of answers, among participants: around 35% agreed and 55% disagreed. Component 8 includes statements 28, 29 and 32. These statements are related to the experience of taking a Pap smear test. Among participants, the test is perceived as painful (38%) and uncomfortable (49%). Additionally, 19% of the respondents believed that the health professionals performing the test are rude to women. Lastly, component 9 included four statements: 34, 35, 36 and 37. Interestingly, these four items were added to the instrument as result of the discussion with SDS public health experts. However, on average, 83% of the participants disagreed with the statements.

There is a relationship between the scores of three constructs of the CHBM, severity, motivation and barriers, and the poverty level of the participant. Kruskal–Wallis tests show that there are statistically significant differences in the scores of severity (*p-value* < 0.001), health motivation (*p-value* < 0.0028) and barriers (*p-value* < 0.001) between the three levels of poverty. Additionally, the Dunn tests show that participants at the higher level of poverty have lower health motivation score (*p-value* < 0.001), highest severity score (*p-value* < 0.001) and higher barriers score (*p*-value < 0.001). There is no statistically significant difference among the scores of the other two groups of participants. Pairwise comparisons for the nine components lead to similar conclusions regarding the poverty levels. Additional file 1: Table S3 presents the *p-values* for the Kruskal–Wallis test and the pairwise comparisons, using the Dunn test.

### Variables affecting no-show probability

This section presents the LASSO regression results. We report the odds ratios (5th percentile, 95th percentile and average) for the 100 experiments. While Table 3 presents the results for the HBM survey, Table 4 presents the results for the patient and appointment characteristics. Both tables present the results of the same LASSO model. To model the outcome, a value of one is assigned to those patients attending their appointments. Therefore, higher odds ratios (ORs) mean lower no-show probabilities. This model has a good discriminatory power and its results are not sensitive to the sample. The average AUROC score is 0.79 with a standard deviation of 0.004.

**Table 3:** LASSO regression results: Health Beliefs Model survey

Category	*N*	Statement	Odds ratio disagree			Odds ratio neutral		
			95th	5th	Average	95th	5th	Average
Susceptibility	1	It is likely that I will get cervical cancer in the future	0.90	0.74	0.82	1.00	0.90	0.98
Susceptibility	3	I feel I will get cervical cancer sometime during my life	0.72	0.60	0.66	0.84	0.65	0.73
Severity	7	I am afraid to think about of cervical cancer	0.99	0.83	0.93	1.25	1.00	1.05
Severity	9	Cervical cancer would threaten a relationship with my husband	1.39	1.16	1.26	1.28	1.03	1.15
Severity	10	If I had cervical cancer my whole life would change	1.29	1.27	1.27	1.19	1.00	1.04
Benefits	13	Maintaining good health is extremely important to me	0.69	0.45	0.54	0.99	0.79	0.92
Benefits	17	If cervical cancer was found at a regular cytology its treatment would not be so bad	1.00	0.92	0.98	1.22	1.02	1.09
Motivation	21	I exercise at least 3 times a week for my health	1.00	0.95	0.99	0.97	0.81	0.89
Motivation	22	I have regular health check-ups even when I am not sick	0.87	0.75	0.80	1.11	1.00	1.02
Barriers	23	I am afraid to have a Pap smear test for fear of a bad result	1.42	1.21	1.31	1.80	1.32	1.52
Barriers	28	Undergoing a Pap smear test is too painful	1.36	1.11	1.23	1.12	1.00	1.02
Barriers	29	Health professionals performing Pap smear tests are rude to women	0.98	0.82	0.93	1.39	1.08	1.22
Barriers	30	I have other problems in my life which are more important than having a Pap smear test	1.00	0.86	0.95	1.59	1.13	1.32
Barriers	32	Undergoing a Pap smear test is too uncomfortable	1.58	1.27	1.42	1.26	1.00	1.05
Barriers	33	I think that having a regular Pap smear test is required only if one has an active sexual life	1.55	1.28	1.42	1.15	1.00	1.02
Barriers	35	Preparing for cytology can be inconvenient for me	1.00	0.83	0.95	1.48	1.04	1.22

**Table 4:** LASSO regression results: Patient and appointment characteristics

Variable		Odds Ratio for attendance probability		
		5th	95th	Average
*Age*				
	[25, 30)	0.83	0.98	0.93
	[30, 59)	0.81	0.99	0.94
	[59, 64)	1.62	2.29	1.96
	> 64	1.00	1.00	1.00
*Poverty*				
	High	0.66	0.85	0.74
	Medium	1.00	1.21	1.08
	Low	1.00	1.00	1.00
*Lead time*				
	< 14	2.95	3.78	3.24
	[14, 27)	4.00	5.40	4.63
	[27, 39)	1.76	2.38	2.01
	> 39	1.00	1.00	1.00
*Month*				
	January	0.95	1.00	0.99
	February	1.16	1.02	1.06

There is a relationship between the CHBM constructs and the no-show probability. Table 3 summarises the ORs for the possible answers to 16 items of the survey. The other 21 items were found not to have a good predictive value for the attendance levels. Participants with higher perceived susceptibility are more likely to keep their appointments. Those who disagree with statements 1 and 3 have OR of 0.82 and 0.66, respectively. Additionally, patients with lower health motivation and perceived benefits are less likely to attend. The average ORs range from 0.54 to 1.09 for benefits and from 0.80 to 1.02 for health motivation.

Perceived severity and barriers affect the no-show probability. Patients who disagree with *being afraid to think about cervical cancer* are less likely to attend (OR 0.93). Surprisingly, those who do not worry about specific personal consequences of the disease have lower no-show probabilities (OR 1.26 and 1.27). Additionally, patients are more likely to attend if they are not afraid to have the test (OR 1.31), do not think that the test is painful (OR 1.23) or uncomfortable (1.42) and do not believe that the testing is only required for patients with an active sexual life (1.42). Lastly, patients have lower no-show probabilities if they are neutral to statements 29, 30 and 35.

We also find a relationship between patient and appointment characteristics and the attendance probability. As can be seen in Table 4, the age and the poverty level of the patient affect her attendance rate. The older the patient, the more likely they are to keep their appointment. Additionally, patients in the highest level of poverty have lower attendance probabilities. Table 4 also shows that reducing lead times might lead to better attendance levels. ORs range from 1 to 4.63 when the lead time is varied. Lastly, as the survey was conducted between January and February 2020, the model is not able to find possible seasonal patterns on the attendance rates. The ORs for February appointments are only slightly higher than the ones for January.

### Improving prediction accuracy

**Fig. 2:**
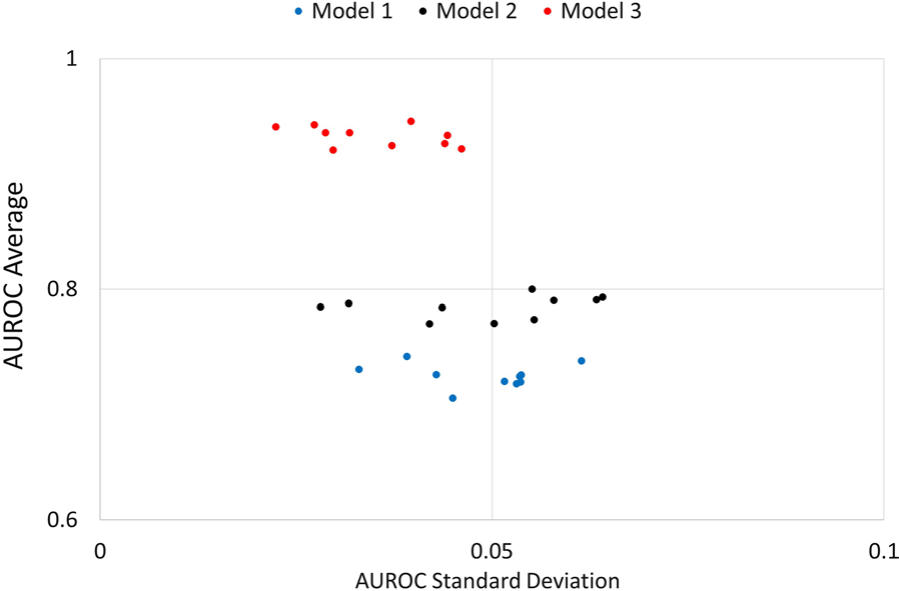
Model performance

In this section, we assess the performance of the three modelling approaches to predict individual attendance probabilities. Figure 2 summarizes the results of 300 experiments. Each point in the graph represents the average and standard deviation of the AUROC score for a group of ten experiments. Model 1 predicts the attendance probability using only patient and appointment variables presented in Table 1. Model 2 includes the same variables and the results from the HMB survey. Lastly, model 3 follows a sequential approach, combining data from 23,384 Pap smear appointments and the survey results.

Assessing patient beliefs towards cervical cancer screening adds value to the prediction process. By using the survey results, it is possible to increase the average AUROC score from 0.71 to 0.79. Arguably, collecting and processing this information is expensive. However, these results improve the understanding of the no-show phenomena and could be used to inform the design of interventions to increase attendance levels. This is particularly relevant in the context of hard-to-reach patients. Additionally, the performance of model 3 shows that it is possible to train one model with administrative data (routinely collected) and select a representative sample of patients to assess their beliefs. This strategy increases the AUROC score up to 0.9.

## Discussion

Compared to other studies using Guvenc’s scale, our results suggest that hard-to-reach women from Bogotá have lower perceived susceptibility [22, 37–40], higher perceived severity [21, 22, 37–42], higher perceived benefits [38–44] and lower perceived barriers [21, 38, 41, 42, 45, 46], towards cervical cancer screening. Recent reviews concluded that these beliefs have been less researched in Latin America [47, 48]. However, we identified among our participants three beliefs that have hampered the implementation of cervical cancer screening programs in other countries of the region. Firstly, for 32% of our participants there is a relationship between cancer history in the family and the susceptibility of developing cervical cancer [49, 50]. Secondly, 28% of the women believe that undergoing a Pap smear test is not required if one does not have an active sexual life [49, 51, 52]. Lastly, 41% of the surveyed patients think that a cervical cancer diagnosis might threaten the relationships with their husbands, boyfriends or partners [51, 53, 54].

Our regression results also confirm what has been found in previous research, in LMIC contexts outside Latin America. In Bogotá, patients are more willing to undergo a Pap smear test if they perceive themselves at risk of developing cervical cancer or understand the benefits of the screening program. Similarly, perceived susceptibility was associated with higher uptake rates in Ghana [55], Ethiopia [39] and Iran [56]. Additionally, higher perceived benefits were found to encourage screening behaviours in Nepal [41], Ghana [55] and Ethiopia [57]. In a recent review, Simbar et al. [58] concluded that training-based interventions are able to modify perceived susceptibility and benefits, leading to behavioural changes. Therefore, education among participants with higher no-show risk in Bogotá should aim at increasing perceived susceptibility.

We also find that poverty affects patients’ beliefs and attendance probabilities. Participants in the most severe level of poverty have lower perceived health motivation, higher perceived severity, higher perceived barriers and are less likely to keep their appointments. The relationship between poverty levels and cervical cancer screening behaviour [52, 59–62], or no-show rates [63–66], has been widely documented [30]. However, little has been discussed about the differences in beliefs among women suffering different levels of poverty. Targeting marginalized communities with tailored interventions could improve screening uptake [2, 8, 67]. Therefore, our results suggest the need to develop new information material for lower income patients in Bogotá.

Cancer worries decrease attendance probability. The underlying assumption of the CHBM is that perceived susceptibility acts as an enabler for protective health behaviours. Indeed, several studies have found that perceived severity is associated with better cervical cancer screening uptake rates [20, 37, 41, 68, 69]. However, our results show that participants who believe that a cervical cancer diagnosis would threaten their relationships (41%) or change their whole life (74%), and participants who are afraid of a bad result (36%) are less likely to attend. Recent research has theorized that there is a difference between general (about the disease) and specific (about the consequences) cancer worries [70]. In this context, it is possible that while worrying about developing cancer motivates early diagnosis behaviours, some specific worries about the consequences act as deterrents to screening attendance [71, 72]. Our results highlight the need to develop education campaigns to address misconceptions about the disease mortality and treatment.

There is a potential for improving attendance rates among hard-to-reach women in Bogotá by decreasing lead times. ORs range from 1 to 4.63 (IC 4–5.4) when the lead time is decreased. This relationship has been previously found in other no-show studies for healthcare appointments [73–75]. Further, while offering timely access to screening services is a key component in implementation success [76, 77], access problems are one of the main barriers towards cervical cancer screening in Latin America [51]. It has been argued that in poorly-resourced systems, cytology-based screening programs are less effective than using a combination of different types of test [78, 79]. Our results highlight the need to conduct an economic evaluation of alternatives to strength the cervical cancer screening program in Bogotá. For example, including HPV testing and self-sampling have shown positive impacts in Argentina, Brazil and Mexico [80–83].

The main limitations of this study are related to the sample. First, we aimed at assessing beliefs among hard-to-reach women in Bogotá. Therefore, sampling among ACS participants is considered to be a good strategy. However, we are not able to draw conclusions about other relevant groups in the city. Further research on women outside the program could also inform public policy. Second, data were collected at the end of the home visit. Consequently, it is not possible to quantify the impact of the basic training provided by ACS community workers among our participants. However, we believe that the information provided by this assessment can be used to strengthen the program and ultimately improve health outcomes. Lastly, data were collected between January and February 2020. In Bogotá, the first confirmed case of COVID-19 was reported in March 2020 and restrictions on social distance were adopted two weeks later. Data collection was completed before the public became aware of the pandemic so we are confident this did not influence responses, but it is not possible to draw any conclusions on how the (widely available) information on the virus may subsequently have affected the health-seeking behaviours of our study population.

## Conclusion

Our methodological approach has the potential to improve the cost-effectiveness of behavioural interventions to increase screening uptake among hard-to-reach women in any setting. Generally, behavioural strategies aimed at the whole population are not cost-effective [12, 84, 85]. Further, using mass interventions such as phone or text reminders might ignore the underlying reasons for the no-show behaviour among hard-to-reach populations [86, 87]. Therefore, by accurately predicting individual attendance probabilities, it is possible (and financially sustainable) to design tailored interventions for marginalized communities in low-resourced settings. More importantly, for each cohort of patients the model can be used to predict individual attendance probabilities and classify patients into different intervention groups. By doing so, costly behavioural interventions can be reserved for those with higher no-show risk. In this context, scores of the beliefs assessment can be used to select the most appropriate behavioural approach for each group. We have also shown that, following a sequential approach, it is possible to identify patients with higher no-show risk by exploiting a combination of routinely-collected data and a sample-based beliefs assessment. In Bogotá, interventions for younger patients living in extreme poverty should be prioritized. Additionally, educational campaigns should be designed to address misconceptions about the disease mortality and treatment.

## Supplementary Information

**Additional file 1.** Descriptive statistics.

**Additional file 2.** Instrument validation.

## Data Availability

The datasets generated during and analysed during the current study are not publicly available due to patient confidentiality. However, they are available from SDS (subject to further ethical approval) on reasonable request. Please contact the corresponding author in the first instance.

## Abbreviations

ACS: *Acciones colectivas en salud*
AUROC: Area under the receiver operating characteristics
CHBM: Champion’s revised health belief model
HBM: Health belief model
KMO: Kaiser–Meyer–Olkin test
LASSO: Least absolute shrinkage and selection operator
SDS: *Secretaría distrital de salud*
