# Supplementary material for: Improving intervention design to promote cervical cancer screening among hard-to-reach women: assessing beliefs and predicting individual attendance probabilities in Bogotá, Colombia
Source: BMC Womens Health. 2022 Jun 7;22:212. doi: 10.1186/s12905-022-01800-3 (PMC9172610; doi:10.1186/s12905-022-01800-3)
Supplement: Supplementary file 2 — Additional file 2. Instrument validation. [file 12905_2022_1800_MOESM2_ESM.docx]

### **Instrument validation**

Table 1 summarizes the results for the item reliability tests. For each statement, mean, standard deviation, coefficient of variation, correlation with the other items in the same category and Cronbach’s alpha coefficient (if the item is deleted) are provided. On the one hand, statements in the Barriers category have the highest average variability. Coefficients of variation for this category range from 0.63 to 0.83, with an average of 0.76. Items 25, 27, 30, 31, 34 and 35 have coefficients of variation above 0.8. On the other hand, the perceived benefits are more similar among the participants. Coefficients of variation for this category range from 0.19 to 0.36, with an average of 0.24. Additionally, items 13 and 15 have coefficients of variation below 0.2. Average coefficients of variation for susceptibility, severity and motivation are 0.65, 0.44 and 0.51, respectively.

In order to identify poorly-functioning items, we adopted the criteria defined by Guvenc et al. [20]. Statements with a correlation below 0.3 with category scores, or showing an increase greater than 0.1 in the Cronbach’s coefficient, if deleted, should be removed. Only six items have a correlation below 0.4: three severity items (9, 10 and 11) and three benefits items (12, 17 and 19). However, as shown in Table 1 they all are above 0.3. Additionally, Cronbach’s coefficients for each category are: Susceptibility 0.74, Severity 0.74, Benefits 0.73, Health motivation 0.62 and Barriers 0.82. As can be seen in Table 1, there is no statement that increases the coefficient of its category above 0.1 when it is removed. Therefore, all the items met the inclusion criteria.

Table 1: Item reliability

| **Category** | No. | Statement | Mean | SD | Mean/SD | Correlation* | Alpha |
| --- | --- | --- | --- | --- | --- | --- | --- |
| **Susceptibility** | 1 | It is likely that I will get cervical cancer in the future | 3.47 | 1.83 | 0.53 | 0.57 | 0.73 |
| **Susceptibility** | 2 | My chances of getting cervical cancer in the next few years are high | 2.72 | 1.83 | 0.67 | 0.66 | 0.68 |
| **Susceptibility** | 3 | I feel I will get cervical cancer some time during my life | 2.78 | 1.84 | 0.66 | 0.63 | 0.70 |
| **Susceptibility** | 4 | I feel I will get cervical cancer some time during my life because I have family history of cancer | 2.45 | 1.82 | 0.74 | 0.47 | 0.78 |
| **Severity** | 5 | The thought of cervical cancer scares me | 4.26 | 1.46 | 0.34 | 0.57 | 0.68 |
| **Severity** | 6 | When I think about cervical cancer I feel worried | 4.25 | 1.44 | 0.34 | 0.61 | 0.67 |
| **Severity** | 7 | I am afraid to think about of cervical cancer | 4.32 | 1.41 | 0.33 | 0.62 | 0.67 |
| **Severity** | 8 | Problems I would experience with cervical cancer would last a long time | 3.54 | 1.72 | 0.49 | 0.44 | 0.71 |
| **Severity** | 9 | Cervical cancer would threaten a relationship with my husband, boyfriend or partner | 2.99 | 1.83 | 0.61 | 0.32 | 0.74 |
| **Severity** | 10 | If I had cervical cancer my whole life would change | 4.14 | 1.55 | 0.37 | 0.38 | 0.72 |
| **Severity** | 11 | If I developed cervical cancer, I would not live longer than 5 years | 2.83 | 1.75 | 0.62 | 0.31 | 0.74 |
| **Benefits** | 12 | I want to discover health problems early | 4.59 | 1.15 | 0.25 | 0.39 | 0.70 |
| **Benefits** | 13 | Maintaining good health is extremely important to me | 4.76 | 0.88 | 0.19 | 0.51 | 0.68 |
| **Benefits** | 14 | I look for new information to improve my health | 4.38 | 1.32 | 0.30 | 0.40 | 0.70 |
| **Benefits** | 15 | I feel it is important to carry out activities which will improve my health | 4.75 | 0.89 | 0.19 | 0.54 | 0.67 |
| **Benefits** | 16 | Having regular Pap smear tests will help to find changes to the cervix, before they turn into cancer | 4.67 | 0.98 | 0.21 | 0.46 | 0.69 |
| **Benefits** | 17 | If cervical cancer was found at a regular Pap smear test its treatment would not be so bad | 4.11 | 1.48 | 0.36 | 0.34 | 0.73 |
| **Benefits** | 18 | I think that having a regular Pap smear test is the best way for cervical cancer to be diagnosed early | 4.66 | 1.02 | 0.22 | 0.46 | 0.69 |
| **Benefits** | 19 | Having regular Pap smear tests will decrease my chances of dying from cervical cancer | 4.52 | 1.21 | 0.27 | 0.35 | 0.71 |
| **Category** | No. | Statement | Mean | SD | Mean/SD | Correlation* | Alpha |
| **Motivation** | 20 | I eat well-balanced meals for my health | 4.04 | 1.59 | 0.39 | 0.43 | 0.53 |
| **Motivation** | 21 | I exercise at least 3 times a week for my health | 3.06 | 1.84 | 0.60 | 0.46 | 0.48 |
| **Motivation** | 22 | I have regular health check-ups even when I am not sick | 3.45 | 1.83 | 0.53 | 0.40 | 0.56 |
| **Barriers** | 23 | I am afraid to have a Pap smear test for fear of a bad result | 2.66 | 1.85 | 0.70 | 0.42 | 0.88 |
| **Barriers** | 24 | I am afraid to have a Pap smear test because I don’t know what will happen | 2.59 | 1.86 | 0.72 | 0.48 | 0.88 |
| **Barriers** | 25 | I don’t know where to go for a Pap smear test | 2.08 | 1.70 | 0.81 | 0.50 | 0.87 |
| **Barriers** | 26 | I would be ashamed to lie on a gynaecologic examination table | 2.39 | 1.82 | 0.76 | 0.55 | 0.87 |
| **Barriers** | 27 | Undergoing a Pap smear test takes too much time | 1.91 | 1.55 | 0.81 | 0.62 | 0.87 |
| **Barriers** | 28 | Undergoing a Pap smear test is too painful | 2.72 | 1.87 | 0.69 | 0.51 | 0.87 |
| **Barriers** | 29 | Health professionals performing Pap smear tests are rude to women | 2.04 | 1.59 | 0.78 | 0.54 | 0.87 |
| **Barriers** | 30 | I have other problems in my life which are more important than having a Pap smear test | 1.83 | 1.48 | 0.81 | 0.56 | 0.87 |
| **Barriers** | 31 | I am too old to have a Pap smear test regularly | 1.73 | 1.43 | 0.83 | 0.57 | 0.87 |
| **Barriers** | 32 | Undergoing a Pap smear test is too uncomfortable | 3.09 | 1.93 | 0.63 | 0.44 | 0.88 |
| **Barriers** | 33 | I think that having a regular Pap smear test is required only if one has an active sexual life | 2.26 | 1.77 | 0.78 | 0.52 | 0.87 |
| **Barriers** | 34 | My religion does not allow me to undergo a Pap smear test | 1.52 | 1.23 | 0.81 | 0.62 | 0.87 |
| **Barriers** | 35 | Preparing for a Pap smear test can be inconvenient for me | 1.61 | 1.31 | 0.82 | 0.65 | 0.87 |
| **Barriers** | 36 | Undergoing a Pap smear test can cause problems with my partner | 1.54 | 1.22 | 0.79 | 0.61 | 0.87 |
| **Barriers** | 37 | I am too young to have a Pap smear test regularly | 1.38 | 1.02 | 0.74 | 0.46 | 0.88 |

* Item- rest correlation

Table 2 presents the results of the principal component analysis (the Kaiser Mayer Olkin test result is 0.86 and the *p-value* for Bartlett’s test is below 0.001). Nine components are identified with an eigenvalue greater than one, accounting for 57.7% of the variability. While no item has a loading value greater than 0.3 outside the intended category, five items did not meet the criteria for inclusion in any of the components (25, 26, 27, 30 and 31). All these items belong to the Barriers category of the instrument. Finally, for three categories, more than one component can be identified: Severity (two components), Benefits (two components) and Barriers (three components). As can be seen in Table 2, the three Barriers components explain 30% of the variability.

Table 2. Results from the principal component analysis.

| Component | Category | Statement | Eigenvalue | Proportion* |
| --- | --- | --- | --- | --- |
| 1 | Susceptibility | 1, 2, 3, 4 | 2.05 | 5.5% |
| 2 | Severity | 5, 6, 7 | 2.57 | 6.9% |
| 3 | Severity | 8, 9, 10, 11 | 1.08 | 2.9% |
| 4 | Benefits | 12, 13, 14, 15 | 1.54 | 4.2% |
| 5 | Benefits | 16, 17, 18, 19 | 1.42 | 3.8% |
| 6 | Motivation | 20, 21, 22 | 1.22 | 3.3% |
| 7 | Barriers | 23, 24 | 1.48 | 4.0% |
| 8 | Barriers | 28, 29, 32 | 3.82 | 10.3% |
| 9 | Barriers | 34, 35, 36, 37 | 6.16 | 16.7% |

### *Proportion of the variability explained by each component
